# Classifying the Acquisition Sequence for Brain MRIs Using Neural Networks on Single Slices

**DOI:** 10.7759/cureus.22435

**Published:** 2022-02-21

**Authors:** Norbert Braeker, Cornelia Schmitz, Natalie Wagner, Badrudin J Stanicki, Christina Schröder, Felix Ehret, Christoph Fürweger, Daniel R Zwahlen, Robert Förster, Alexander Muacevic, Paul Windisch

**Affiliations:** 1 Department of Radiation Oncology, Kantonsspital Winterthur, Winterthur, CHE; 2 Data Science, Propulsion Academy, Zurich, CHE; 3 Radiosurgery, European Cyberknife Center, Munich, DEU; 4 Radiation Oncology, Charité - Universitätsmedizin Berlin, Berlin, DEU; 5 Medical Physics, European CyberKnife Center, Munich, DEU; 6 Stereotaxy and Neurosurgery, University Hospital, Cologne, DEU; 7 Radiosurgery, European CyberKnife Center, Munich, DEU

**Keywords:** deep learning, grad-cam, acquisition sequence, perspective, mri, brain imaging

## Abstract

Background

Neural networks for analyzing MRIs are oftentimes trained on particular combinations of perspectives and acquisition sequences. Since real-world data are less structured and do not follow a standard denomination of acquisition sequences, this impedes the transition from deep learning research to clinical application. The purpose of this study is therefore to assess the feasibility of classifying the acquisition sequence from a single MRI slice using convolutional neural networks.

Methods

A total of 113 MRI slices from 52 patients were used in a transfer learning approach to train three convolutional neural networks of different complexities to predict the acquisition sequence, while 27 slices were used for internal validation. The model then underwent external validation on 600 slices from 273 patients belonging to one of four classes (T1-weighted without contrast enhancement, T1-weighted with contrast enhancement, T2-weighted, and diffusion-weighted). Categorical accuracy was noted, and the results of the predictions for the validation set are provided with confusion matrices.

Results

The neural networks achieved a categorical accuracy of 0.79, 0.81, and 0.84 on the external validation data. The implementation of Grad-CAM showed no clear pattern of focus except for T2-weighted slices, where the network focused on areas containing cerebrospinal fluid.

Conclusion

Automatically classifying the acquisition sequence using neural networks seems feasible and could be used to facilitate the automatic labelling of MRI data.

## Introduction

While research on the application of deep learning on magnetic resonance imaging (MRI) to detect, classify, and contour brain tumors has been conducted for several years and achieved impressive initial results in many cases, the amount of research that has been translated into clinical application is limited [1-3].

Among the reasons often cited for this phenomenon are the lack of publicly available datasets, and homogenous datasets acquired on few scanners, which limit the ability of a neural network to generalize to images from different scanners and the effort required to label the data [4,5].

A factor that plays another important role but is less frequently accounted for in the design of research projects is the difference between the carefully curated data used to train, validate, and test the neural networks in research projects, and the less structured, real-world data that is acquired as part of routine clinical practice.

In research projects, models are oftentimes trained with only those combinations of perspective and acquisition sequence that have the most potential to enable the model to complete the respective task or answer the primary research question, such as T1-weighted, contrast-enhanced axial slices to detect and segment brain metastases [3].

Real-world data, however, are far less consistent. An MRI study consists of different series that vary between different radiology departments and practices as well as for different indications, and not all of them are relevant for the task that the model is trained to perform [6].

In addition, the different sequences do not follow a consistent naming pattern since different manufacturers use their own terminology, leading to different denominations (e.g. gradient echo being denoted with a variety of terms such as “GRE,” “FFE,” “FE,” “GE,” etc.), which is especially problematic for the development of device agnostic software solutions, i.e., applications that are supposed to be able to work with MR images from different scanners and practices.

If a neural network could be trained to distinguish perspective as well as acquisition sequence and only pass the relevant series to another neural network that was trained to make the actual predictions, this could be used to facilitate the application of highly specific neural networks to real-world data.

In addition, one could use a network like the following to develop more sophisticated deep learning workflows.

Just as radiologists do not limit themselves to looking only at the series that is the most promising to answer a question but consult other perspectives and sequences if needed, a model could potentially enhance its performance by mimicking said behavior. If a neural network could be trained to distinguish perspective and acquisition sequence and send each series of an MRI to a specialized network that is only trained to handle images of this particular combination, the predictions of each specialized network could be passed to an ensemble method that weighs them and provides a final prediction for the whole study.

To assess the feasibility of this approach, we train neural networks of different complexities to classify the acquisition sequence of MR images using only a limited number of slices from a publicly available dataset.

## Materials and methods

**Figure 1:**
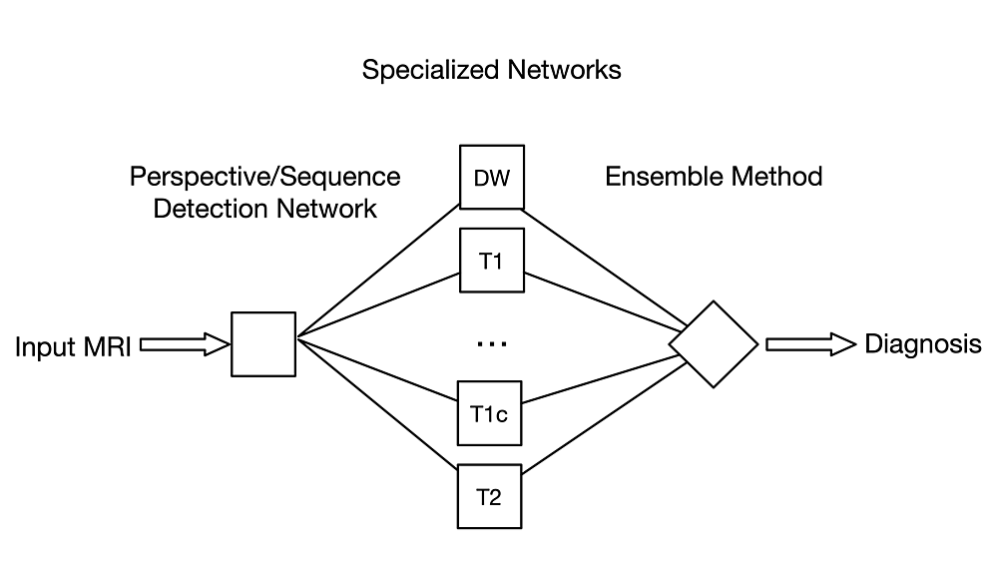
Possible application of a neural network for sequence classification in a deep learning workflow For each MRI study, a neural network detects the different acquisition sequences and passes the corresponding series to a specialized neural network that was trained to perform predictions (e.g. detect pathologies) on this particular sequence. The predictions based on each series are then passed to an ensemble method (possibly a neural network), which takes them into account to create a final prediction for the whole study. DW, diffusion-weighted; T1, T1-weighted; T1c, T1-weighted with contrast enhancement; T2, T2-weighted

Herein, we train and test a neural network to distinguish perspective and acquisition sequence so that it can function in the aforementioned approach, as illustrated in Figure 1.

All imaging data were handled in accordance with institutional policies, and an approval by an institutional review board was obtained for the analysis of the brain metastases dataset (Institutional Review Board of the Ludwig Maximilian University of Munich, Munich, Germany, project 20-437). Written informed consent for the analysis of anonymized clinical and imaging data was obtained from all patients.

To assess the robustness of the approach across architectures of varying complexity, three pretrained convolutional neural networks (ResNet-18, ResNet-34, and ResNet-50) were used as part of a transfer learning approach and retrained on MRI slices that were labeled with the respective acquisition sequence (T1-weighted without contrast enhancement, T1-weighted with contrast enhancement, T2-weighted, and diffusion-weighted) [7].

A total of 140 MRI slices containing macroscopic tumors from 52 patients were created by randomly selecting patients from The Cancer Genome Atlas (TCGA) glioblastoma (GBM) dataset publicly provided as part of The Cancer Imaging Archive (TCIA) [8,9].

In addition, 113 slices were used as training data (24 T1-weighted without contrast enhancement, 29 T1-weighted with contrast enhancement, 36 T2-weighted, and 24 diffusion-weighted).

For the internal validation, 27 slices were used (7 T1-weighted without contrast enhancement, 7 T1-weighted with contrast enhancement, 7 T2-weighted, and 6 diffusion-weighted). To prevent data leakage, the slices of the training and the internal validation cohort were created from different patients.

Data generated or analyzed during the study are available from the corresponding author by request.

Programming was done using Python (version 3.6) and fastai (version 2) as well as PyTorch (version 1.7) libraries. The different ResNet versions pretrained on ImageNet were provided via the PyTorch library. All codes used for training and validating the networks are provided as a supplemental file.

Data augmentation was performed with the fastai RandomResizedCrop (minimum scale = 0.9) and aug_transforms functions, with the latter providing rotation, zoom, and changes to brightness as well as contrast. All images were resized to 224 x 224 pixels prior to inputting them to the ResNet.

Training was performed using the fastai fine_tune method for a total of 45 epochs with a variable learning rate and training for the first five epochs while frozen. Flattened cross-entropy loss was used as the loss function and Adam as the optimizer [10].

**Figure 2:**
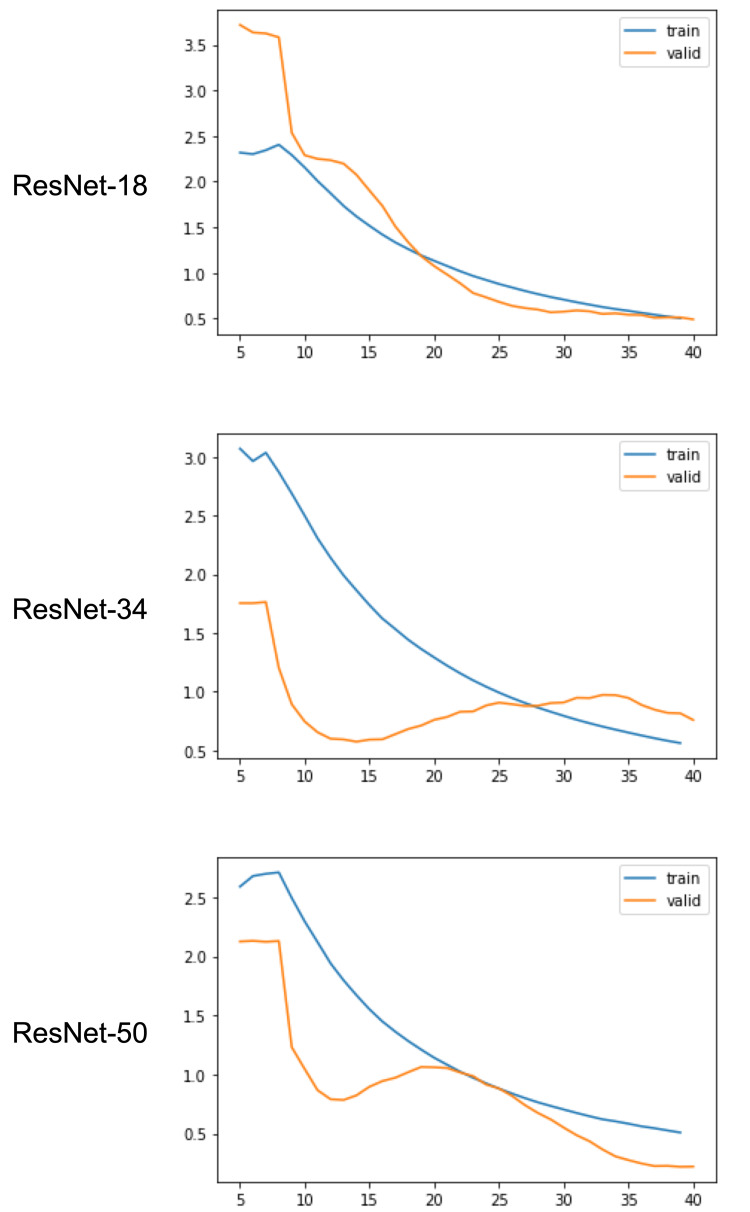
Training and (internal) validation loss by training epoch for the different neural networks

Training and validation loss for the three networks are shown in Figure 2.

The networks then underwent an external validation of their categorical accuracy on 600 slices (150 T1-weighted without contrast enhancement, 150 T1-weighted with contrast enhancement, 150 T2-weighted, and 150 diffusion-weighted) created from 273 patients acquired on various scanners provided by the European Radiosurgery Center in Munich. While the MRIs of the patients from the validation set contained either brain metastases or meningiomas, the slices were created randomly so that some slices contained tumor while others did not.

In a final step, we implemented Grad-CAM to identify regions that the predictions of the networks were based on [11]. Grad-CAM provides visual explanations for the decision of a neural network by highlighting areas that were important to the network when making the decision.

## Results

All models improved to a categorical accuracy between 0.80 and 0.89 on the internal validation data during the training period.

**Figure 3:**
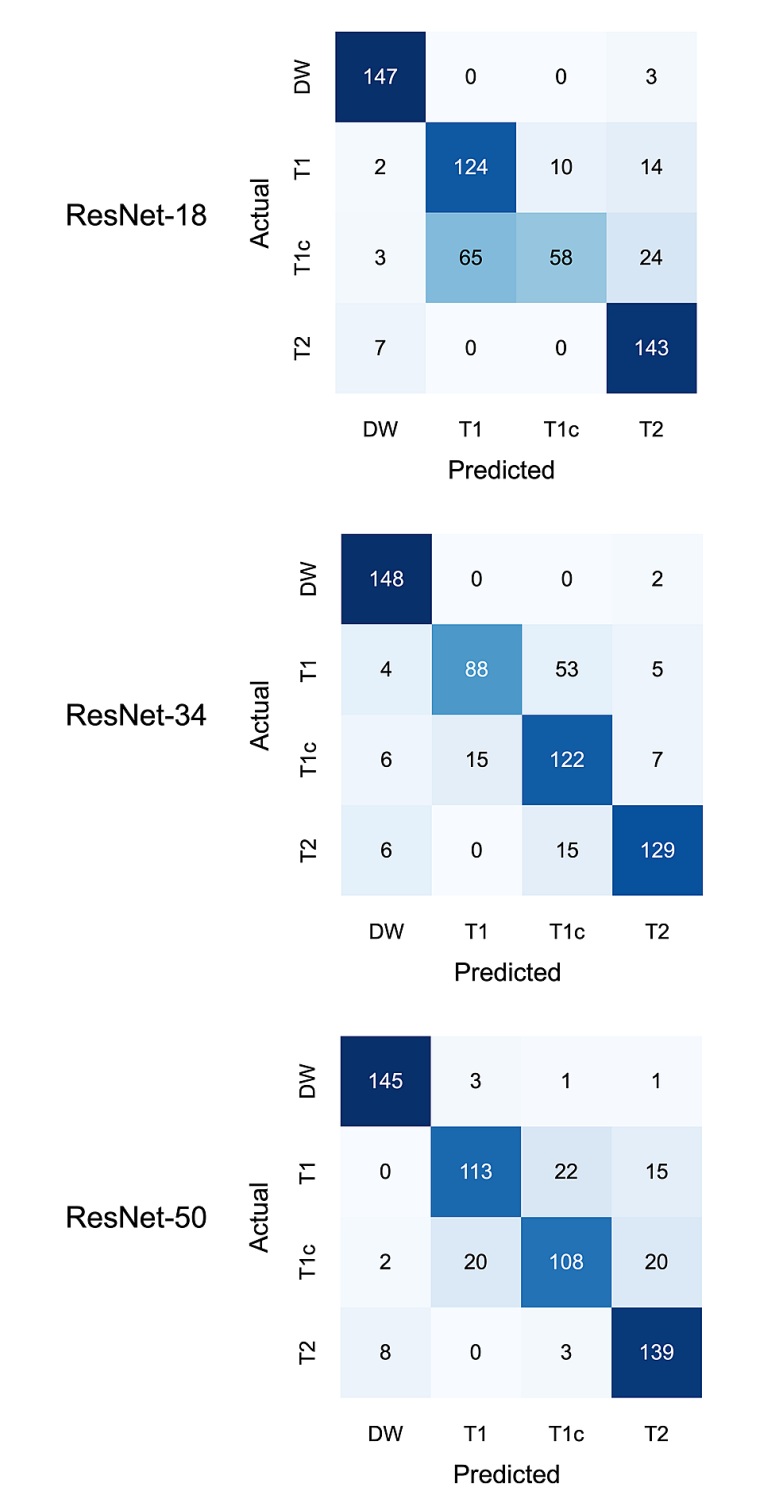
Confusion matrices Confusion matrices for the performance of the neural networks on the validation set consisting of 600 images (150 images per class) from 273 patients. DW, diffusion-weighted; T1, T1-weighted; T1c, T1-weighted with contrast enhancement; T2, T2-weighted

The external validation of the models resulted in categorical accuracy of 0.79 for ResNet-18, 0.81 for ResNet-34, and 0.84 for ResNet-50. The confusion matrices of the performance on the test set are provided in Figure 3.

While the simpler architectures struggled to differentiate T1-weighted images without contrast enhancement from T1-weighted images with contrast enhancement, this was less the case for the ResNet-50. However, differentiating between those two classes remained the most challenging problem, while diffusion-weighted images were almost never confused for one of the other classes.

Figure 3 shows the implementation of Grad-CAM for predictions of the ResNet-50 on 16 correctly-classified slices (4 T1-weighted without contrast enhancement, 4 T1-weighted with contrast enhancement, 4 T2-weighted, 4 diffusion-weighted) from 16 different patients. While the network seemed to highlight cerebrospinal fluid (CSF) when looking at a T2-weighted image, the pattern for the T1-weighted images was less consistent. When classifying diffusion-weighted images, the network was more likely to consider the background instead of the brain itself compared to images belonging to the other classes.

## Discussion

The solid categorical accuracy on the external validation data given the very limited number of images that were used for training suggests that differentiating the acquisition sequence of MR images using neural networks is feasible.

Additional studies with larger datasets are required to investigate the performance when adding more classes to accommodate different acquisition sequences (perfusion-weighted, magnetic resonance angiography, etc.).

The performance on the heterogeneous external validation cohort can be considered especially promising with regard to the fact that the TCGA-GBM MRIs that were used for training had been acquired prior to 2011, while the MRIs of the external test set had, for the most part, been acquired more recently. Even though the MRIs from the different eras exhibit some differences regarding their visual appearance, the networks showed a significant ability to generalize.

While a categorical accuracy of 0.84 is not perfect and trying to improve the accuracy by adding more data can be considered, the Digital Imaging and Communications in Medicine (DICOM) standard metadata of MR images can be used to achieve an almost perfect performance in practice, even with a sub-optimal accuracy: even though the denomination of the different series is not standardized, images of a given patient belonging to the same series can be identified by having identical DICOM tags, e.g., for “Scanning Sequence” [12]. When the neural network evaluates all slices of a given patient that belong to the same series and conducts a “majority vote” by assigning all of them to the most frequent prediction it made, the limited number of slices that would have received a wrong prediction receive the prediction of the majority instead, which should almost always be correct, given the relatively high categorical accuracy.

If the categorical accuracy on a single slice could be increased, e.g. by adding more training data, one could reduce the number of slices from a series that need to be sampled to get a correct prediction for the whole series. This would reduce the required computational power and thus the time for the classification, which is an important consideration regarding the application in routine clinical practice.

Since this prototype used only 140 slices from 52 patients and rather old MRIs, the chance of increasing the performance by increasing the number of slices and patients as well as using a combination of older, publically available datasets with more recent institutional data seems realistic.

The literature on the application of deep learning for the automatic classification of MRI sequences is sparse. Noguchi et al. conducted a study by retraining AlexNet and GoogLeNet on 384 slices from 78 patients. While they describe an even greater accuracy of 0.93-1.0 depending on the location of the slice and the pathology of the patient, the lack of an external validation cohort makes it difficult to assess the generalizability of their approach [13].

**Figure 4:**
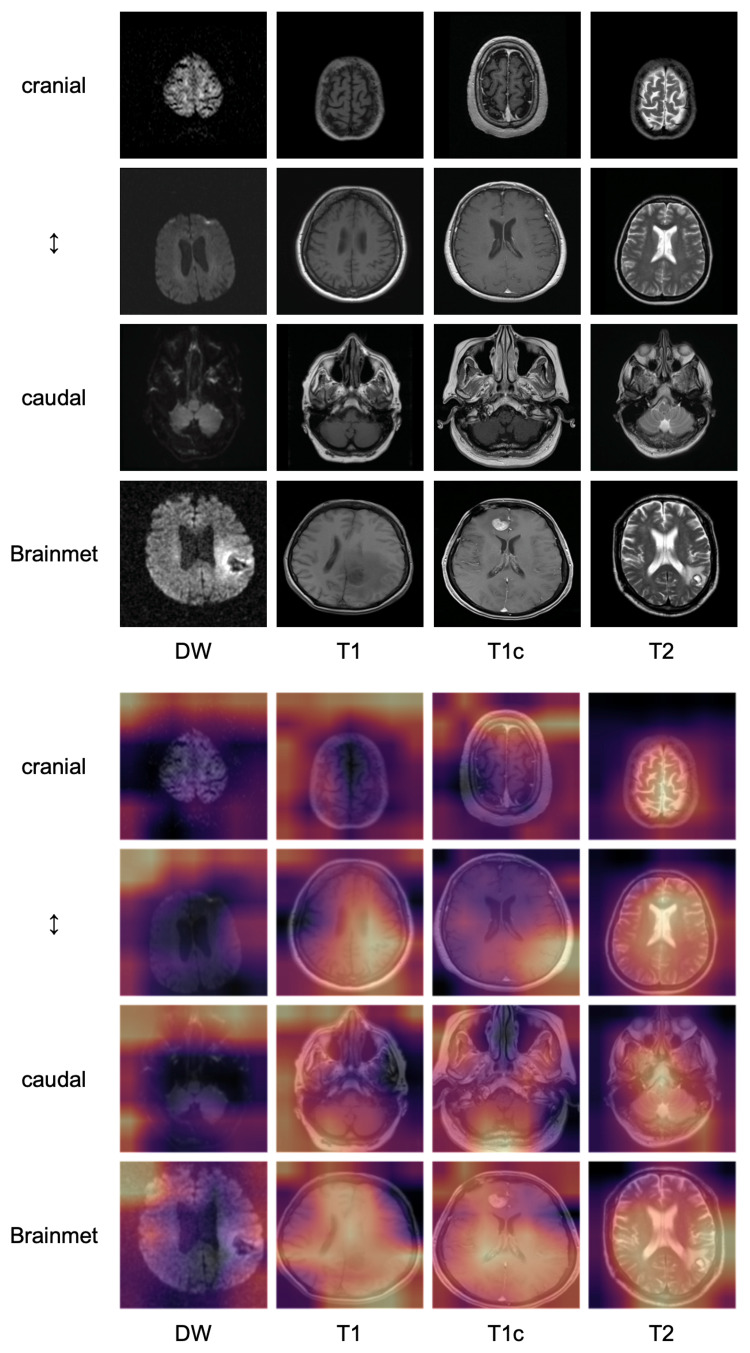
Implementation of Grad-CAM Implementation of Grad-CAM for ResNet-50 on 16 correctly classified images from 16 different patients highlighting the areas of an image the predictions of the model are based on. The top three rows show slices without macroscopic tumors on different levels of the brain, while the bottom row shows slices containing tumors on a similar level. When looking at T2-weighted images, the network tends to highlight areas of the image that contains cerebrospinal fluid. DW, diffusion-weighted; T1, T1-weighted; T1c, T1-weighted with contrast enhancement; T2, T2-weighted

As the training slices were initially created for another purpose and not sequence prediction, all slices contained macroscopic tumors. We therefore had the suspicion that the performance could suffer drastically on slices from the validation set that did not contain tumor, especially for contrast-enhanced slices, where, at least to human readers, the enhancing tumor, if present, is a very prominent feature of an image that could potentially have distracted the network from recognizing more subtle features of contrast enhancement during training. Indeed, the differentiation between enhanced and non-enhanced T1-weighted slices was a problem for the simpler architectures, while the ResNet-50 was less affected. When looking at the Grad-CAM predictions for slices containing macroscopic brain metastases (Figure 4), it seems that ResNet-50 is indeed making its predictions while mostly ignoring the tumor, even on the T1-weighted slice with contrast enhancement.

The fact that the network focused on areas containing CSF when looking at T2-weighted images is unsurprising considering that the bright signal of the CSF is an easy way to differentiate those images from the other three classes.

A question that could be of interest in the future is how well a neural network trained with images from one region of the body, in case of this study the brain, is able to perform when trying to classify images from another region, for example, the abdomen. In addition, it will be interesting to examine the regions that the predictions of the neural networks are based on when they are trained on a greater number of images, images from different body regions, or images containing more classes. While a clear pattern could only be found for T2-weighted images with this prototype, training the neural networks with larger datasets might lead to more consistent patterns.

Limitations of this proof-of-concept study include the limited number of classes and the fact that the pathologies that were present on the slices of the validation set, if any, were only two tumor entities. It is unclear how other pathologies such as intracranial hemorrhage could affect the performance of the network. In addition, an improved performance of the networks could probably be achieved by adapting the amount of training epochs to the complexity of the respective network. For the sake of consistency, all models were trained for the same number of epochs in this study.

## Conclusions

In conclusion, the automatic classification of the acquisition sequence using neural networks for a limited number of classes was feasible in this study with limited training data and might enable developing more sophisticated deep learning radiology workflows in the future. Further research is warranted to assess the performance when increasing the number of classes and its use for research and clinical applications.
